# Transcriptome Analysis Reveals that Exogenous Melatonin Confers *Lilium* Disease Resistance to *Botrytis elliptica*

**DOI:** 10.3389/fgene.2022.892674

**Published:** 2022-06-14

**Authors:** Xuehua Xie, Yu Han, Xi Yuan, Man Zhang, Ping Li, Aiqin Ding, Jia Wang, Tangren Cheng, Qixiang Zhang

**Affiliations:** ^1^ Beijing Key Laboratory of Ornamental Plants Germplasm Innovation & Molecular Breeding, Beijing Forestry University, Beijing, China; ^2^ National Engineering Research Center for Floriculture, Beijing Forestry University, Beijing, China; ^3^ Beijing Laboratory of Urban and Rural Ecological Environment, Beijing Forestry University, Beijing, China; ^4^ Engineering Research Center of Landscape Environment of Ministry of Education, Beijing Forestry University, Beijing, China; ^5^ Key Laboratory of Genetics and Breeding in Forest Trees and Ornamental Plants of Ministry of Education, Beijing Forestry University, Beijing, China; ^6^ School of Landscape Architecture, Beijing Forestry University, Beijing, China

**Keywords:** melatonin, disease resistance, *Botrytis elliptica*, transcriptome analysis, MAPK cascades

## Abstract

Leaf blight, caused by *Botrytis elliptica* (Berk.) Cooke, is a devastating disease that limits the production of *Lilium* in China and in other countries worldwide. Numerous studies have indicated that plants have evolved sophisticated and effective signal transduction and defense-related pathways in response to pathogen invasion. Recently, particular attention has been given to the action(s) of melatonin in plants in response to biotic stress, and the role of melatonin in plant–pathogen interactions has also been discussed. In this study, RNA-seq was applied to analyze the transcriptomic changes in *Lilium* leaves that were pre-treated and post-treated with melatonin after *B. elliptica* infection for 0, 12, 24, 36, and 72 h and then compare those changes with those of the control. Treatment with exogenous melatonin and infection with *B. elliptica* caused differential expression of a large number of genes in *Lilium* leaves. KEGG pathway analysis showed that, after melatonin treatment, the defense-related DEGs were mainly enriched in plant–pathogen interactions, plant hormone signal transduction, MAPK signaling pathways, phenylpropanoid biosynthesis, and phenylalanine metabolism. RT–qPCR was used to verify the expression changes of 12 DEGs, the results of which were consistent with the RNA-seq analysis results. The expression of DEGs related to the MAPK pathway were significantly different between the MB group and the HB group, suggesting that, via the MAPK signaling cascade, melatonin may play a role in the disease resistance of *Lilium* to *B. elliptica*. This study provides a new perspective and information for molecular-based breeding of *Lilium* disease resistance.

## 1 Introduction

*Lilium* is constantly affected by bacteria, viruses, and fungi, resulting in heavy economic losses during its growth. Specifically, leaf blight caused by *Botrytis elliptica* (Berk.) Cooke is a devastating disease that causes enormous losses to cut flower production and gardening applications under hot and heavy rainfall conditions. Understanding the molecular basis or resistance mechanism against pathogens is important for the sustainable production and breeding of new varieties. Studies on the mechanisms of plant disease resistance have shown that plants have evolved complex mechanisms to sense invading pathogens and employ proper responses accordingly downstream of effector-triggered immunity (ETI) or pathogen-triggered immunity (PTI) activation (Chisholm et al., 2006; Jones and Dangl, 2006; Boller and Felix, 2009; Dodds and Rathjen, 2010; Cook et al., 2015). In addition to three primary defense hormones, other plant hormones, such as abscisic acid (ABA), brassinosteroids (BRs), and cytokinins (CKs), are also vital resistance-related compounds that activate complex phytohormone signaling networks involved in plant defense against *Botrytis cinerea* (AbuQamar et al., 2017).

To date, the majority of related studies have focused the role of melatonin (N-acetyl-5-methoxytryptamine) since it was discovered in vascular plants in 1995 (Dubbels et al., 1995). Due to its wide-ranging functions in plants, as a pleiotropic signaling molecule, melatonin plays important roles in the regulation of plant growth; development; and defense against various environmental stresses, such as drought, salt, cold, heat, and heavy metals (Arnao and Hernández-Ruiz, 2014; Byeon and Back, 2014; Nawaz et al., 2015; Wei et al., 2015; Arnao and Hernandez-Ruiz, 2018; Gu et al., 2021; Tiwari et al., 2021). In addition, previous studies have suggested that exogenous melatonin improves plant resistance to pathogen infection and shows its efficacy against devastating fungal pathogens such as *Diplocarpon mali* in apple (Yin et al., 2013), *Phytophthora infestans* in potato (Zhang et al., 2017), *Podosphaera xanthii* in cucurbits (Mandal et al., 2018). Mycelial growth of phytopathogenic fungi (*Colletotrichum gloeosporioides* and *Colletotrichum acutatum*) which caused severe anthracnose in *Capsicum annum* were significantly reduced 76 and 71% after a 100 μM concentration of phytomelatonin treatment (Ali et al., 2021).

Melatonin is a powerful antioxidant that can remove excess ROS and RNS and indirectly activate both enzymatic and non-enzymatic antioxidant systems under biological stress, acting as a signal molecule for elevated defense response against bacterial infection. The application of melatonin has been found to enhance the production of SA, nitric oxide (NO) and hydrogen peroxide (H_2_O_2_) in plants infected with pathogens (Lee et al., 2014). Studies have implicated melatonin in the induction of plant resistance to pathogens alone or in conjunction with the defense-related hormones SA, NO and H_2_O_2_ (Shi et al., 2015a; Shi et al., 2015b; Qian et al., 2015), indirect crosstalk of melatonin-phytohormone will help the plants to cope with the pathogen attack and a working model of melatonin-mediated plant resistance has been proposed (Zhao et al., 2019; Tiwari et al., 2021). In addition, by affecting lignin and gossypol synthesis genes involved in the phenylpropanoid and gossypol pathways, melatonin enhances cotton immunity to *Verticillium wilt* (Li et al., 2019). An increasing number of researchers have implicated melatonin in the regulation of the immune signaling network and interactions between plants and pathogens (Shi et al., 2015a; Li et al., 2016; Wei Y. et al., 2018). Study of melatonin spray in alleviating powdery mildew infection in watermelon. Demonstrated that melatonin played a positive role in enhancing plant resistance by enhancing the expression of PTI and ETI response-related genes in watermelon (Mandal et al., 2018), however, the direct effect of melatonin on promoting PTI and ETI is not well explored. Researches show that signaling with respect to melatonin-mediated innate immunity in plants occurs through various *MAPKKK* kinases within MAPK signaling cascades (Lee and Back, 2016; Lee and Back, 2017). MAPK signaling cascades are important signaling modules and play important roles in plant growth; development; and adaptation to environmental stress, such as cold, drought, and to pathogens (Banerjee et al., 2020). Following pathogen infection, ROS and NO bursts activate MAPK signaling cascades to phosphorylate downstream targets, including transcription factors, and to promote the synthesis of defense-related enzymes and other antimicrobial compounds that in turn activate cellular responses (Frawley and Bayram, 2020; Zhang and Zhang, 2022). However, it is still unclear whether such a response of melatonin in disease resistance is universal across plant species, and the mechanism underlying melatonin-mediated disease resistance is unknown.

In this study, we conducted an RNA sequencing (RNA-seq) analysis of *Lilium* genes that were differentially expressed in response to melatonin and involved in resistance to *B. elliptica*. The results therefore reveal the metabolic mechanism underlying melatonin-mediated resistance of *Lilium* to *B. elliptica*.

## 2 Materials and Methods

### 2.1 Plant Materials and Fungal Culture Conditions

The oriental hybrid *Lilium* cultivar “Sorbonne” was selected for this study. Bulbs were planted in pots (17 cm in diameter) containing peat and pearlite (1:1) as a growth substrate. The pots were placed in a greenhouse at Beijing Forestry University under a 12 h day/night photoperiod at 25/22°C. *B. elliptica* isolated from diseased *Lilium* leaves was grown on potato dextrose agar media for 7 days at 25°C. Mycelial discs (1 cm in diameter) were subsequently obtained with a leather punch for inoculation.

### 2.2 Treatment and Experimental Design

The roots of plants growing for 45 days were treated with 0, 0.02, 0.2, 2, or 20 mM melatonin (100 ml per plant) for 6 and 10 days (once every 2 days). Melatonin (Sigma–Aldrich, St. Louis, MO, United States) solutions were prepared by dissolving the soluble materials in ethanol followed by dilution with Milli-Q water. After melatonin pretreatment, six leaves from the middle of plants were inoculated with *B. elliptica*, and the area of each lesion was measured at 36, 72 and 120 h after inoculation. Six plants were used per treatment, and this process was repeated three times. Based on the percentage of lesion area covering the total area of the *Lilium* leaves, the disease severity was assessed on the basis of a 5-point scale: 0, no visible symptoms; 1, symptoms <5%; 2, symptoms = 5%–25%; 3, symptoms = 25%–50%; 4, symptoms >50%. The disease index was calculated by assessing 36 leaves and then calculated with the following formula:

$$\text{Disease index}=\frac{\sum \left(\text{number of leaves for different grade }\times \text{ grade}\right)}{\text{Total assessed leaves }\times \;7}$$

The experiment for transcriptome sequencing consisted of two parts: pre-treatment and post-treatment. In the pretreatment part, *Lilium* plants were treated with 100 ml of a 2 mM melatonin solution by irrigating the roots once every other day with the solution, for a total of five times. Control plants were treated with 0 mM melatonin, and then the leaves in the middle of the plants were inoculated with *B. elliptica*. The plants inoculated with *B. elliptica* were cultivated in a growth chamber at 25°C under a 16 h light/8 h dark photoperiod, and samples were obtained at five time points (0, 12, 24, 36, and 72 h) after inoculation from the inoculated sites for transcriptome sequencing. In the posttreatment part, *Lilium* plants were inoculated with *B. elliptica* for 36 h, and then the inoculated plants were treated with 100 ml of 2 and 0 mM melatonin solutions by irrigating the roots with those solutions. Leaf samples from the inoculated sites were obtained for transcriptome sequencing at 36 h after treatment. Each treatment included three biological replications, and each replication comprised 12 plants. All the samples used for transcriptome sequencing were frozen immediately in liquid nitrogen and stored at −80°C.

### 2.3 Enzyme Extraction and Activity Assays

Leaf samples from plants pre-treated with 2 and 0 mM melatonin were obtained at five time points (0, 12, 24, 36, 48, and 72 h) after inoculation with *B. elliptica*. The harvested samples were rapidly frozen in liquid nitrogen and stored at −80°C for the phenylalanine ammonia lyase (PAL) and catalase (CAT) assays. A PAL test kit (Nanjing Jiancheng Bioengineering Institute, A137-1-1) and CAT assay kit-visible light (Nanjing Jiancheng Bioengineering Institute, A007-1-1) were used for enzyme extraction and activity assays. The reaction mixture, operation process and enzyme activity were prepared, performed, and calculated, respectively, according to the instructions of the kits.

### 2.4 RNA Extraction, Library Preparation, and Illumina Sequencing

Total RNA was extracted using an RNAprep Pure Plant Kit (Tiangen, Beijing, China) as described by the manufacturer’s protocol for each RNA-seq sample, and RNA quality was detected via 1% agarose gel electrophoresis. The RNA degradation, purity, concentration, and integrity were measured using a Nanodrop™ 2000 spectrophotometer (Thermo Fisher Scientific, United States), Qubit RNA Assay Kit with Qubit Fluorometer 2.0 (Life Technology, Carlsbad, CA, United States), and an Agilent Bioanalyzer 2100 system (Agilent Technologies, CA, United States). Sequencing libraries were constructed using a NEB Next^Ⓡ^ Ultra™ RNA Library Prep Kit for Illumina^®^ (NEB, Beverly, CA, United States). Library preparation sequencing was performed on an Illumina HiSeq 4000 platform, with 150 bp paired-end reads.

### 2.5 Sequence Assembly and Annotation

Quality control analysis was conducted for raw reads obtained from the sequencing data to identify high-quality sequencing data and clean reads. The Trinity v2.4.0 platform (with the parameters K-mer = 25 and group pair distance = 250) was used to stitch and assemble clean reads (Grabherr et al., 2011). Redundant sequences were removed from the unigene sequences with TGICL software, and sequences with lengths as long as possible were assembled. Fragments per kilobase of exon per million mapped fragments (FPKM) were used to standardize the read counts of each gene. BLASTX searches were performed based on the information housed in the following databases: the National Center for Biotechnology Information (NCBI) nonredundant (Nr) protein sequence database (e-value = 1e^−5^), the NCBI nonredundant nucleotide (Nt) sequence database (e-value = 1e^−5^), the SwissProt manually annotated and reviewed protein sequence database (e-value = 1e^−5^), the Protein family (Pfam) database (e-value = 0.01), the EuKaryotic Orthologous Groups (KOG)/Clusters of Orthologous Genes (COG) database (e-value = 1e^−3^), the Gene Ontology (GO) database (standardized classification for gene function; e-value = 1e^−6^), and the Kyoto Encyclopedia of Genes and Genomes (KEGG) database (gene product functions and metabolic pathways; e-value = 1e^−10^).

### 2.6 Differentially Expressed Gene Analysis

DEG analysis was performed using the DESeq R package. To evaluate the genes that were significantly expressed between two samples, genes with an adjusted *p* value (q-value) < 0.05 and an |log_2_(fold change) FPKM| > 2 were defined as differentially expressed. The GOseq R package (1.10.0) was used for GO enrichment analysis, with a false discovery rate (FDR) < 0.01, and KOBAS (v2.0.12) was used for KEGG pathway enrichment analysis, with FDR of <0.01 (http://kobas.cbi.pku.edu.cn/). Venn diagrams, bubble maps, and heatmaps were constructed using the online data analysis platform Omicstudio tools (https://www.omicstudio.cn/tool/6).

### 2.7 Quantitative Real-Time PCR Analysis

To validate the RNA-seq results of the gene expression levels reflected by the FPKM values, RT–qPCR was performed using a 7500 Real-Time PCR System (Applied Biosystems, CA, United States) and a SYBR R Premix Ex Taq™ Kit (TaKaRa, Tokyo, Japan). Total RNA was extracted and reverse-transcribed into first-strand cDNA using a PrimeScript™ RT Reagent Kit with gDNA Eraser (Takara Bio, Inc., Shiga, Japan) following the manufacturer’s protocol. RT–qPCR was performed on a 20 ml reaction mixture including 2 μl of first-strand cDNA, 0.6 μl of forward primer, 0.6 μl of reverse primer, 10 μl of SYBR Premix Ex Taq and 6.8 μl of sterile distilled water under the following reaction conditions: 95°C for 3 min, followed by 40 cycles of 95°C for 10 s, 55°C for 15 s and 72°C for 15 s. The *Lilium* eukaryotic elongation factor 1 (EF1, KJ543461) gene was used as a reference gene for normalization (Liu et al., 2016). Experiments were performed for three independent biological replicates and three technical replicates. The 2^−ΔΔCt^ method was used to calculate the relative expression levels of the selected transcripts (Livak and Schmittgen, 2001). Detailed information on the primer sequences used for RT–qPCR is listed in Supplementary Table S6. The correlation coefficients between the RT–qPCR results and FPKM value were analyzed using SPSS 22.0 software.

## 3 Results

### 3.1 Exogenous Melatonin Enhanced *Lilium* Resistance to *B. elliptica*

**FIGURE 1:**
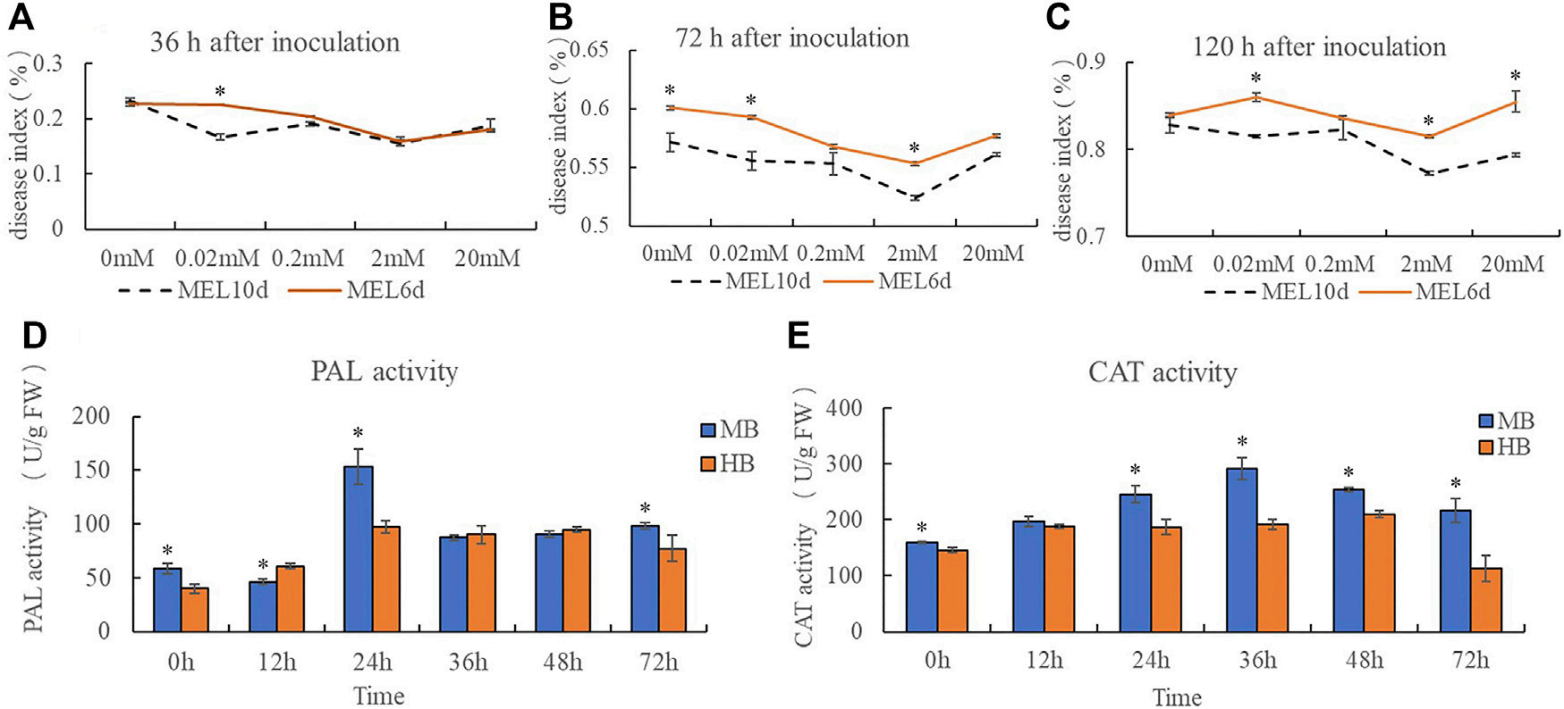
Effect of exogenous melatonin pre-treatment on *Lilium* resistance to *B. elliptica*. **(A)** Effect of melatonin concentration on disease index at 36 h for plants pretreated with 0, 0.02, 0.2, 2 or 20 mM melatonin. MEL6d represents plants were pre-treatment with melatonin for 6 days (once every 2 days), MEL10d represents plants were pre-treatment with melatonin for 10 days (once every 2 days). **(B)** Effect of melatonin concentration on disease index at 72 h for plants pretreated with 0, 0.02, 0.2, 2 or 20 mM melatonin. MEL6d represents plants were pre-treatment with melatonin for 6 days (once every 2 days), MEL10d represents plants were pre-treatment with melatonin for 10 days (once every 2 days). **(C)** Effect of melatonin concentration on disease index at 120 h for plants pretreated with 0, 0.02, 0.2, 2 or 20 mM melatonin. MEL6d represents plants were pre-treatment with melatonin for 6 days (once every 2 days), MEL10d represents plants were pre-treatment with melatonin for 10 days (once every 2 days). **(D)** Effect of exogenous melatonin pre-treatment on Phenylalanine ammonia lyase (PAL) activity of *Lilium* after inoculation. MB represents plants were pre-treatment with 2 mM melatonin and then inoculated with *B. elliptica*; HB represents plants were pre-treatment with Milli-Q water [ethanol/water (v/v) D 1/10,000], and then inoculated with *B. elliptica*. **(E)** Effect of exogenous melatonin pre-treatment on catalase (CAT) activity of *Lilium* after inoculation. MB represents plants were pre-treatment with 2 mM melatonin and then inoculated with *B. elliptica*; HB represents plants were pre-treatment with Milli-Q water [ethanol/water (v/v) D 1/10,000], and then inoculated with *B. elliptica*. Data represent mean ± SE of triplicate assays. The line charts were generated based on IBM SPSS Statistics 20. The “*” represents the significant differences.

A disease index was calculated for plants inoculated with *B. elliptica* after treatment with 0, 0.02, 0.2, 2, or 20 mM melatonin. The results indicate that pre-treatment with exogenous melatonin improved the resistance of *Lilium* plants to *B. elliptica*. The addition of 0.02, 0.2, 2 or 20 mM melatonin alleviated blotch damage to varying degrees compared with that of plants pre-treated with 0 mM melatonin (Figures 1A–C). The 2 mM melatonin concentration was used for subsequent analyses. Compared with plants treated with melatonin for 6 days, plants treated with melatonin for 10 days presented a lower disease index. PAL activity and CAT activity first decreased but then increased during the process of pathogen infection. Moreover, the PAL activity of plants pre-treated with 2 mM melatonin was significantly higher than that of the plants in the control group at 0 and 24 h after inoculation (Figure 1D), and the CAT activity of plants pre-treated with 2 mM melatonin was significantly higher than that of plants in the control group at 24, 36 and 72 h after inoculation (Figure 1E), suggesting that melatonin might act as a regulator to enhance *Lilium* resistance against *B. elliptica*.

### 3.2 Summary of Transcriptome Sequencing Data From the *Lilium* Hybrid Cultivar “Sorbonne”

Thirty six samples for transcriptome sequencing were obtained from pre-treatment plants at five time points after inoculation (MB 0 h, MB 12 h, MB 24 h, MB 36 h, MB 72 h and HB 0 h, HB 12 h, HB 24 h, HB 36 h, HB 72 h) and post-treatment plants (BEM, BEH), three biological replicates were performed for each treatment. Approximately 298.72 Gb of data from 36 samples was obtained after filtering and quality control measures were performed in this study. The total number of clean reads for each sample ranged from 65,403,890 to 86,281,814, and the clean read percentage was between 98.02 and 98.45%. All the above data and the sequencing quality, represented by the Q20 value, Q30 percentage and GC content for each sample, are shown in Supplementary Table S1. The Q30 percentage was greater than 95%, and the GC content was between 45.65% and 45.88%. The clean reads of each sample were mapped to the reference sequence, and the alignment proportion of each sample was greater than 78%. The quality of the sequencing data met the requirements for assembly. After assembly and the removal of redundancy were performed, 220,215 unigenes were ultimately generated with a total length, average length, N50 and GC content of 187,808,990 bp, 852 bp, 1,428 bp and 46.78%, respectively (Supplementary Table S2).

Then, the annotation information of the unigenes was checked via the Nr, Nt, SwissProt, KOG, KEGG, GO, and Pfam databases (Supplementary Table S3). A total of 122,909 (55.81%) unigenes were annotated in the public databases, and the numbers of annotated unigenes in the seven functional databases were 103,159 (Nr: 46.84%), 48,154 (Nt: 21.87%), 71,635 (SwissProt: 32.53%), 81,270 (KOG: 36.90%), 78,538 (KEGG: 35.66%), 77,926 (GO: 35.39%), and 88,964 (Pfam: 40.40%). The unigenes that matched sequences from the genome of oil palm (*Elaeis guineensis*) accounted for 21.37% of the total annotations, followed by date (*Phoenix dactylifera*), *Asparagus officinalis*, pineapple (*Ananas comosus*) and others.

Unigenes annotated in the Nr database were classified into three major functional categories [biological processes (BPs), cellular component (CCs) and molecular functions (MFs)] after GO mapping and functional characterization (Supplementary Figure S2). The top terms with the most annotated genes in the three functional categories were metabolic process (15,042 genes, BP), cell (13,875 genes, CC), and catalytic activity (20,251 genes, MF). We next identified the biological pathways via the KEGG database associated with the annotated sequences, and all the unigenes were assigned to 136 pathways and five KEGG categories based on the pathway hierarchy. A total of 91,104 coding DNA sequences (CDSs) were detected using TransDecoder. It also revealed 20,806 simple sequence repeats (SSRs) distributed among 17,875 unigenes and predicted 3,013 unigenes encoding transcription factors. All the assembled contigs and data can be found in the NCBI BioProject database under accession number PRJNA799047.

### 3.3 Differential Expression Analysis of the *Lilium* Hybrid “Sorbonne” After Treatment With Exogenous Melatonin and Inoculation With *B. elliptica*

**FIGURE 2:**
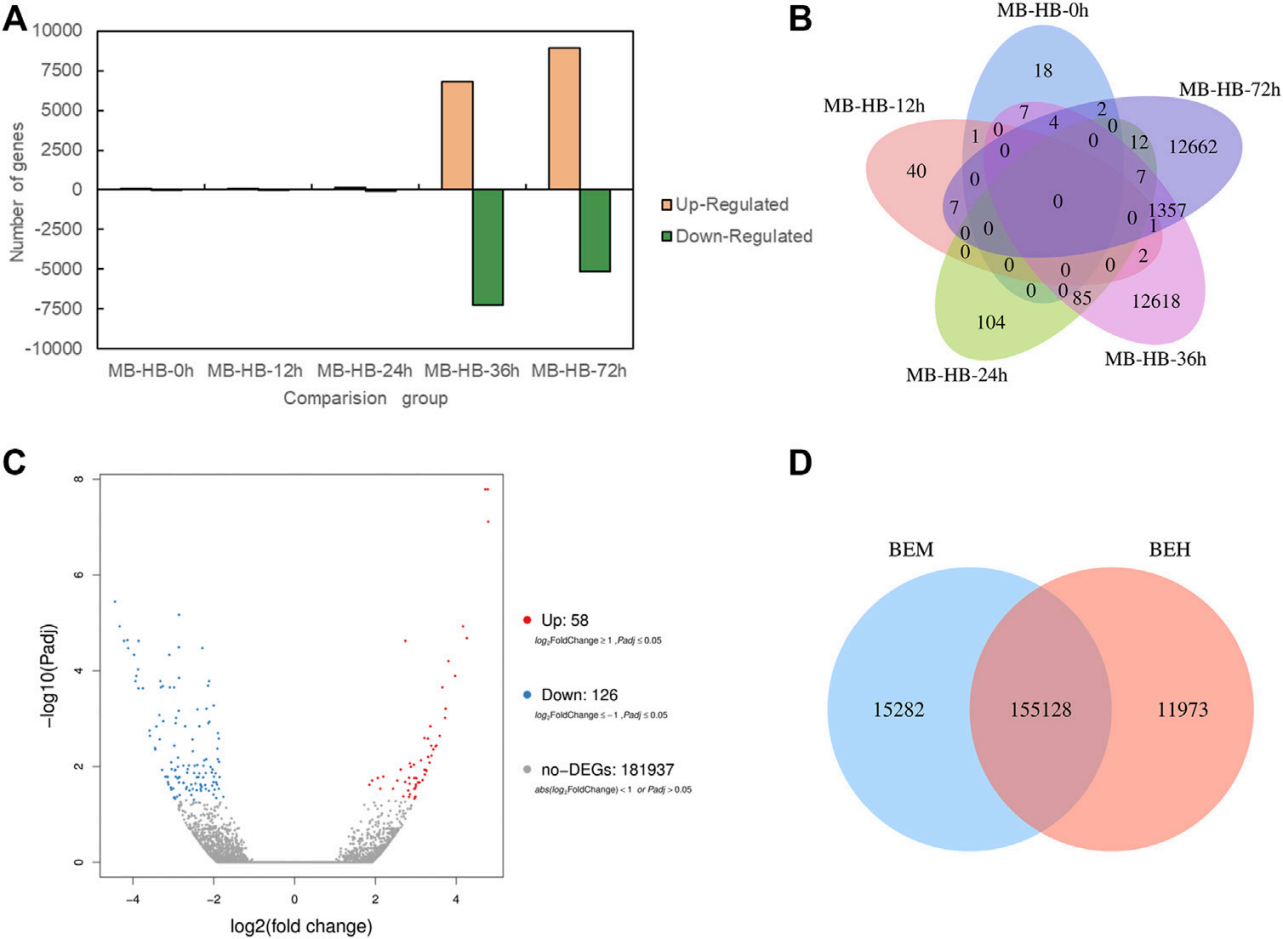
Differentially expressed genes statistics between melatonin treatment group and control group. **(A)** Upregulated and down regulated DEGs (*p*adj < 0.05) statistics of MB group vs. HB group at five points in time after inoculation. MB represents plants were pre-treatment with melatonin and then inoculated with *B. elliptica*; HB represents plants were pre-treatment with Milli-Q water [ethanol/water (v/v) D 1/10,000], and then inoculated with *B. elliptica.* **(B)** Venn diagram of the number of DEGs (*p*adj < 0.05) between MB group vs. HB group. MB represents plants were pre-treatment with melatonin and then inoculated with *B. elliptica*; HB represents plants were pre-treatment with Milli-Q water [ethanol/water (v/v) D 1/10,000], and then inoculated with *B. elliptica.* **(C)** Volcano plot of DEGs (*p*adj < 0.05) between the comparison BEM vs. BEH, BEM represents plants were inoculated with *B. elliptica* for 36 h and then treated with 100 ml melatonin (2 mM/L) for 36 h; BEH represents plants were inoculated with *B. elliptica* for 36 h and then treated with 100 ml Milli-Q water [ethanol/water (v/v). D 1/10,000] for 36 h. **(D)** Venn diagram illustrate the number of DEGs (*p*adj < 0.05) between the comparison BEM vs. BEH, BEM represents plants were inoculated with *B. elliptica* for 36 h and then treated with 100 ml melatonin (2 mM/L) for 36 h; BEH represents plants were inoculated with *B. elliptica* 36 h and then treated with 100 ml Milli-Q water [ethanol/water (v/v) D 1/10,000] for 36 h.

To investigate role of melatonin on the resistance to gray mold of *Lilium*, comparisons were conducted between the melatonin pretreatment group (MB) and the control group (HB) and between the melatonin posttreatment group (BEM) and the control group (BEH) during different infection stages (MB-HB-0 h, MB-HB-12 h, MB-HB-24 h, MB-HB-36 h, MB-HB-72 h, BEM vs. BEH) as shown in Figure 2. Figure 2A displayed upregulated and downregulated DEGs of MB group vs. HB group at five points in time after inoculation, The results showed that the number of upregulated and downregulated DEGs increased significantly at 36 and 72 h after inoculation in the MB vs. HB comparison group. Figure 2B illustrated the number of DEGs overlapping in MB group vs. HB group at five points in time after inoculation, The unoverlapped region represents the unique differential genes in each comparison, The results showed that unique DEGs mainly appeared at 36 and 72 h after inoculation in the MB vs. HB comparison group, suggesting that exogenous melatonin may play a major role in the induction of *Lilium* resistance to *B. elliptica* at 36 and 72 h after inoculation. 58 DEGs were upregulated and 126 DEGs were downregulated in the BEM vs. BEH comparison group (Figure 2C), 15,282 unique DEGs and 11,973 unique DEGs began to present in BEM and BEH group, respectively (Figure 2D).

**FIGURE 3:**
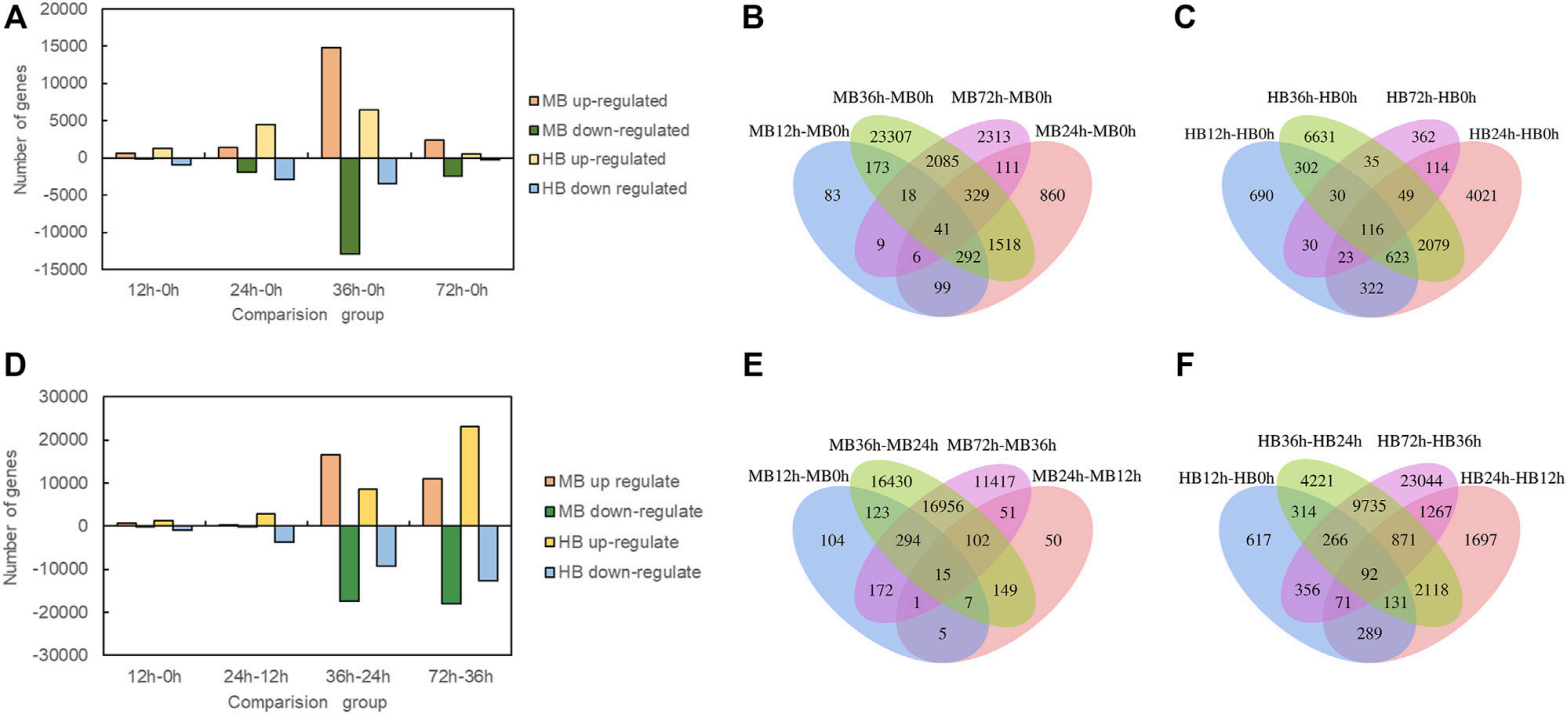
Differentially expressed genes statistics at different time point’s comparison. **(A)** Number of up regulated and down regulated DEGs (*p*adj < 0.05) statistics for four comparisons (12 vs. 0 h; 24 vs. 0 h; 36 vs. 0 h; 72 vs. 0 h) in MB group and HB group. MB represents plants were pre-treatment with melatonin and then inoculated with *B. elliptica*; HB represents plants were pre-treatment with Milli-Q water [ethanol/water (v/v) D 1/10,000], and then inoculated with *B. elliptica*. **(B)** Venn diagram illustrate the number of DEGs in four comparisons (12 vs. 0 h; 24 vs. 0 h; 36 vs. 0 h; 72 vs. 0 h) overlapping in MB group, MB represents plants were pre-treatment with melatonin and then inoculated with *B. elliptica*; **(C)** Venn diagram illustrate the number of DEGs in four comparisons (12 vs. 0 h; 24 vs. 0 h; 36 vs. 0 h; 72 vs. 0 h) overlapping in HB group, HB represents plants were pre-treatment with Milli-Q water [ethanol/water (v/v) D 1/10,000], and then inoculated with *B. elliptica*; **(D)** Number of up regulated and down regulated DEGs (*p*adj < 0.05) statistics for four comparisons (12 vs. 0 h; 24 vs. 12 h; 36 vs. 24 h; 72 vs. 36 h) in MB group and HB group. MB represents plants were pre-treatment with melatonin and then inoculated with *B. elliptica*; HB represents plants were pre-treatment with Milli-Q water [ethanol/water (v/v) D 1/10,000], and then inoculated with *B. elliptica.* **(E)** Venn diagram illustrate the number of DEGs in four comparisons (12 vs. 0 h; 24 vs. 12 h; 36 vs. 24 h; 72 vs. 36 h) overlapping in MB group, MB represents plants were pre-treatment with melatonin and then inoculated with *B. elliptica.* **(F)** Venn diagram illustrate the number of DEGs in four comparisons (12 vs. 0 h; 24 vs. 12 h; 36 vs. 24 h; 72 vs. 36 h) overlapping in HB group, HB represents plants were pre-treatment with Milli-Q water [ethanol/water (v/v) D 1/10,000], and then inoculated with *B. elliptica*.

To investigate the DEGs expressed in *Lilium* in response to *B. elliptica* infection in both the MB group and the HB group, the data in the gene expression libraries corresponding to the five time points were organized into eight pairwise comparisons (12 vs. 0 h, 24 vs. 12 h, 36 vs. 24 h, 72 vs. 36 h; and 12 vs. 0 h, 24 vs. 0 h, 36 vs. 0 h, 72 vs. 0 h) to identify the genes that were differentially expressed during different infection stages as shown in Figure 3. Comprising four points in time with 0 h, the number of upregulated and downregulated DEGs increased significantly in 36 vs. 0 h comparison (Figure 3A), the highest number of unique DEGs was found in 36 vs. 0 h comparison in both the MB group and the HB group (Figures 3B,C). Comprising four points in time with previous points, the number of upregulated and downregulated DEGs of MB group increased significantly in 36 vs. 24 h comparison, while the number of DEGs of HB group was observed in 72 vs. 36 h comparison (Figure 3D), the highest number of unique DEGs was found in 36 vs. 24 h comparison of MB group and 72 vs. 36 h comparison of HB group, respectively (Figures 3E,F), suggesting that exogenous melatonin may accelerate the response of *Lilium* against *B. elliptica*.

To investigate global changes in expression patterns, we performed k-means clustering of the gene expression profiles for orthologous genes in the MB group and the HB group during pathogen infection. We classified the different expression modes into 12 clusters that showed distinctly different expression patterns (Supplementary Figure S1).

### 3.4 Functional Analysis of DEGs in *Lilium* After Treatment With Exogenous Melatonin and Inoculation With *B. elliptica*

DEGs were enriched and analyzed at different time points after pathogen infection with different treatments. GO terms with significant enrichment of DEGs were identified, and GO functional classification analysis of the DEGs between MB group and the HB group (MB-HB-0h, MB-HB-12h, MB-HB-24h, MB-HB-36h, MB-HB-72h, BEM vs. BEH) at five time different points (MB12h vs. MB0h, MB24h vs. MB12h, MB36h vs. MB24h, MB72h vs. MB36h and HB12h vs. HB0h, HB24h vs. HB0h, HB36h vs. HB0h, HB72h vs. HB0h) was performed. In the MB vs. HB comparison group (Supplementary Table S4), the results showed that at 0–24 h after pathogen inoculation, DEGs were significantly enriched in only 11 GO terms: cell wall organization or biogenesis, cellular component organization or biogenesis, structural constituent of cell wall, structural molecular activity, cytidine deaminase activity, and isocitrate lyase activity. At 36 and 72 h after inoculation, DEGs were significantly enriched in 92 GO terms, including carbohydrate metabolic process, chloroplast organization, cutin biosynthetic process, defense response, photosynthesis, peroxiredoxin activity, oxidoreductase activity, and thylakoid membrane. In the BEM vs. BEH comparison group, 16 GO terms were significantly enriched, and three of them were associated with MFs, namely, diacylglycerol O-acyltransferase activity, acylglycerol O-acyltransferase activity, and sequence-specific DNA binding. Thirteen were associated with BPs, including cell response to injury, migration of metal ions, and response to light intensity and ultraviolet light. At different time points, the results showed that, among 301 significantly enriched GO terms in the HB group, 144 (47.8%) were associated with BPs, and 25 (8.3%) were associated with CCs. Among 234 significantly enriched GO terms in the MB vs. HB comparison group, 132 (43.9%) were associated with MFs in the MB group, and 95 (40.6%) were associated with BPs. Sixty (25.6%) were associated with CCs; 79 (33.8%), MFs.

KEGG pathway cluster analysis was conducted between the MB group and the HB group at the five time points to explore the main metabolic pathways in which the DEGs were involved (Supplementary Table S5). The results of KEGG enrichment analysis of the MB vs. HB comparison group showed that 59 metabolic pathways were significantly enriched, which mainly involved energy metabolism, carbohydrate metabolism, amino acid metabolism and synthesis of secondary metabolites. In the BEM vs. BEH comparison group, the DEGs were significantly enriched in 13 metabolic pathways, including circadian rhythm plant (Ko04712); phenylpropanoid biosynthesis (Ko00360); and cutin, suberin and wax biosynthesis (Ko00073). A possible explanation is that exogenous melatonin treatment induced significant changes in the basal metabolism of *Lilium* during pathogen infection, and activity involving 10 metabolic pathways was detected at 0, 12 and 24 h. Activity of the other 49 metabolic pathways was detected at 36 and 72 h after inoculation, which indicated that, within 0–24 h after inoculation, the pathogen was in the colonization stage, did not completely invade the *Lilium* leaves, and did not induce the defense response in *Lilium* leaves. Melatonin may play a role in the response of *Lilium* to pathogen infection and in pathways related to plant disease resistance, such as the peroxisome pathway (Ko04146); MAPK signaling pathway-plant (Ko04016); the phenylpropanoid biosynthesis pathway (Ko00940); plant hormone signal transduction (Ko04075); the phenylalanine metabolism pathway (Ko00360); the cutin, suberin and wax biosynthesis pathway (Ko00073); and fatty acid metabolism pathway (Ko01212). Activity in these pathways was detected within 36 and 72 h after inoculation.

At different points in time, it was found that among all the significantly enriched pathways, the DEGs were mapped to 67 metabolic pathways in the MB group, while the DEGs were mapped to 72 metabolic pathways in the HB group. Significantly enriched pathways related to plant disease resistance mainly included MAPK signaling pathway-plant (Ko04146); phenylpropanoid biosynthesis (Ko00940); phenylalanine metabolism (Ko00360); terpenoid backbone biosynthesis (Ko00900); plant–pathogen interactions (Ko04626); plant hormone signal transduction (Ko04075); cutin, suberin and wax biosynthesis (Ko00073); etc. These pathways might play a key role in *Lilium* resistance to *B. elliptica.*

### 3.5 Transcriptome Analysis of DEGs Involved in the Plant–Pathogen Interaction Pathway, Phenylpropanoid Biosynthesis Pathway, and SA and JA Signaling Pathways During *B. elliptica* Infection

**FIGURE 4:**
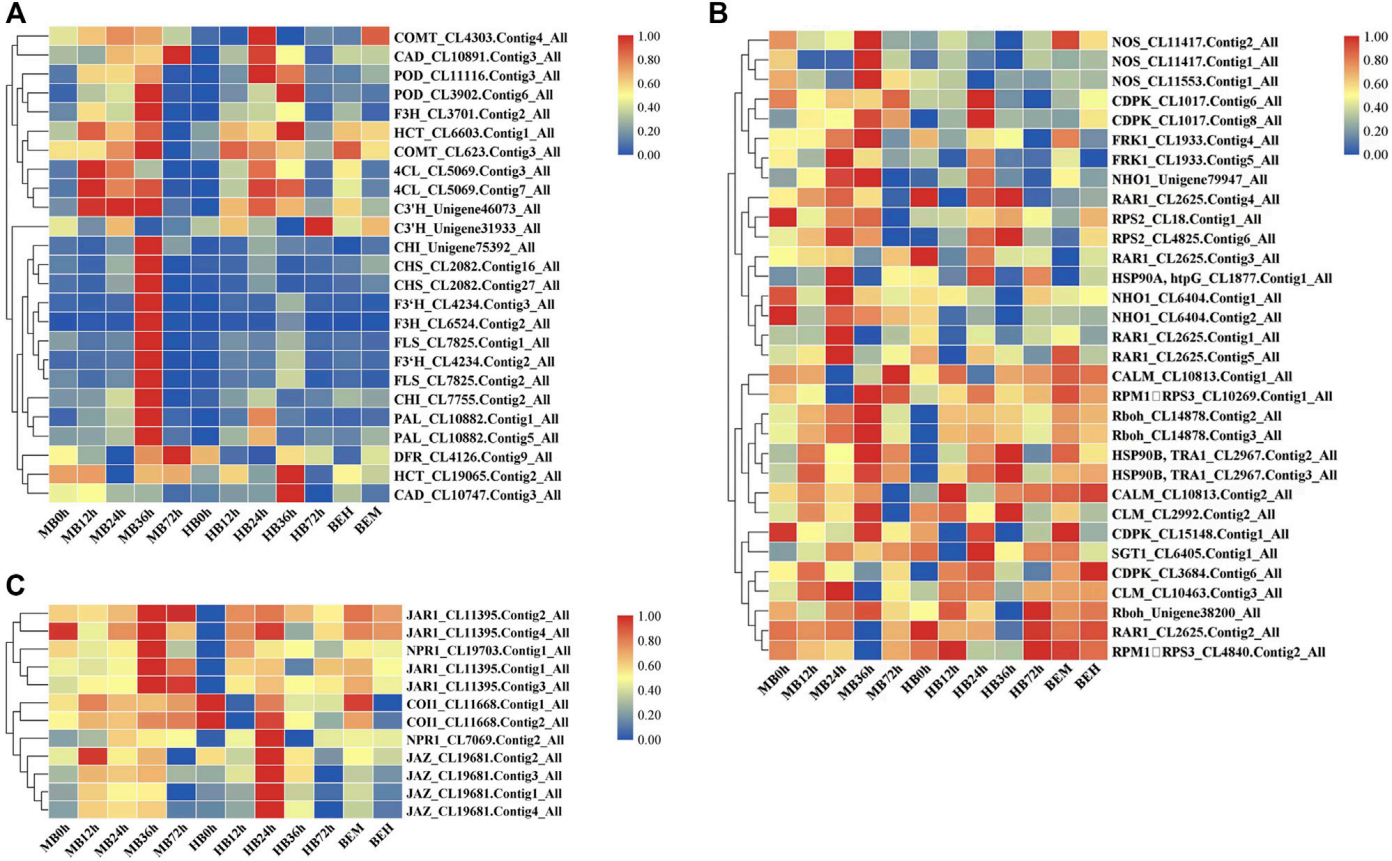
Differential gene expression profiles involved in plant-pathogen interaction pathway, phenylpropanoid biosynthesis pathway, SA and JA signaling pathway during *B. elliptica* infection. **(A)** Gene expression profiles in plant-pathogen interaction pathway. **(B)** Gene expression profiles in phenylpropanoid biosynthesis pathway. **(C)** Gene expression profiles in SA and JA signaling pathway. MB represents plants were pre-treatment with melatonin and then inoculated with *B. elliptica*; HB represents plants were pre-treatment with Milli-Q water [ethanol/water (v/v) D 1/10,000], and then inoculated with *B. elliptica*. BEM represents plants were inoculated with *B. elliptica* for 36 h and then treated with 100 ml melatonin (2 mM/L) for 36 h; BEH represents plants were inoculated with *B. elliptica* for 36 h and then treated with 100 ml Milli-Q water [ethanol/water (v/v) D 1/10,000] for 36 h.

On the basis of the KEGG pathway enrichment data for the 5 time point comparisons after *B. elliptica* inoculation, DEGs that were associated with disease resistance to *B. elliptica* and involved in the phenylpropanoid biosynthesis pathway, plant–pathogen interaction pathway, and plant hormone signal transduction pathway were selected. The gene expression data at the 5 time points is shown in Figure 4. Twenty-five unigenes involved in the phenylpropanoid biosynthesis pathway, including *peroxidase* (*POD*), *caffeic acid 3-O-methyltransferase* (*COMT*), *cinnamyl alcohol dehydrogenase* (*CAD*), *naringenin 3-dioxygenase* (*F3H*), *coumaroyl quinate 3′-monooxygenase* (*C3′H*), *chalcone synthase* (*CHS*), *4-coumarate: CoA ligase* (*4CL*), *PAL*, *cinnamate 4-hydroxylase* (*C4H*), *flavonol synthase* (*FLS*), bifunctional dihydroflavonol 4-reductase (*DFR*), *shikimate O-hydroxycinnamoyl transferase* (*HCT*), *flavonoid 3′-monooxygenase* (*F3′H*), and *chalcone isomerase* (*CHI*), were selected, and most of them were upregulated at 36 h after *B. elliptica* inoculation in the MB group (Figure 4A). Thirty-six unigenes involved in the phenylpropanoid biosynthesis pathway, including *respiratory burst oxidase* (*Rboh*), *calcium-dependent protein kinase* (*CDPK*), *nitric-oxide synthase* (*NOS*), and *LRR receptor-like serine* (*FLS2*), were found to be differentially expressed during *B. elliptica* infection in both the MB group and the HB group (Figure 4C). The plant hormones SA and JA play a major role in disease resistance signaling (Yang et al., 2015). We identified significant expression of the NPR1 and JAR1 genes at 36 h after *B. elliptica* inoculation in the MB group. The F-box protein *coronatine insensitive 1* (*COI1*), working together with *jasmonate zim* (*JAZ*) domain-containing transcriptional repressor proteins, is a key regulator of the JA signaling pathway (Pieterse et al., 2012). The expression of the *COI1* and *JAZ* genes was upregulated at 24 h in the HB group, and spraying exogenous melatonin on infected plants increased *COI1* and *JAZ* gene expression Figure 4B. Detailed information on the DEGs from the KEGG pathway enrichment analysis is listed in Supplementary Table S6.

### 3.6 Role of MAPK Signaling Cascades in the *Lilium* Defense Response Against *B. elliptica*

**FIGURE 5:**
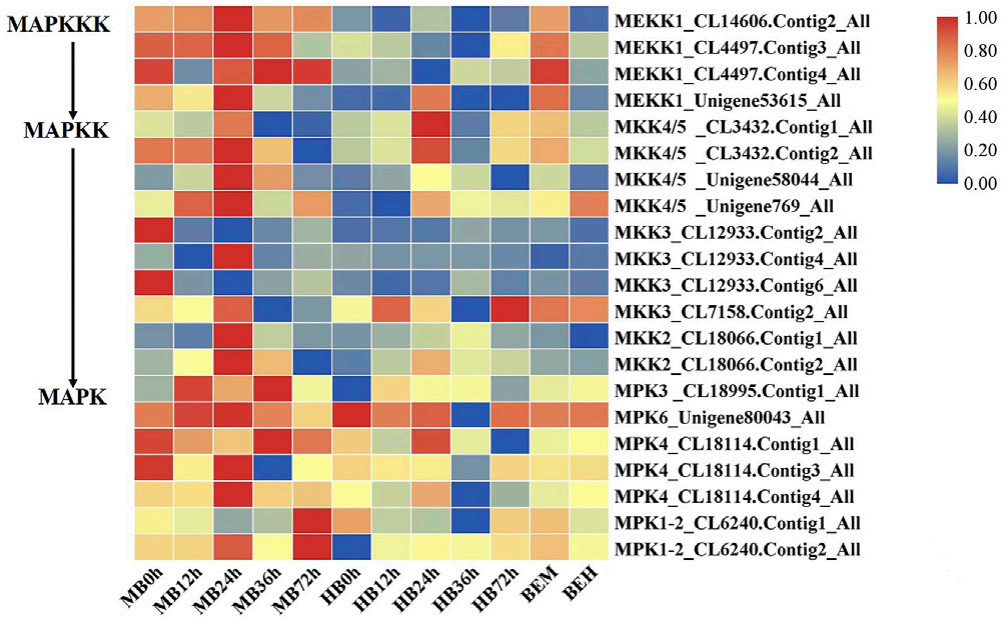
Differential gene expression profiles involved in MAPK Signaling pathway. MB represents plants were pre-treatment with melatonin and then inoculated with *B. elliptica*; HB represents plants were pre-treatment with Milli-Q water [ethanol/water (v/v) D 1/10,000], and then inoculated with *B. elliptica*. BEM represents plants were inoculated with *B. elliptica* for 36 h and then treated with 100 ml melatonin (2 mM/L) for 36 h; BEH represents plants were inoculated with *B. elliptica* for 36 h and then treated with 100 ml Milli-Q water [ethanol/water (v/v) D 1/10,000] for 36 h.

The findings of DEGs involved in MAPK signaling cascades prompted our interests. Plant MAPK signaling cascades play indispensable roles in plant defense against external pathogen attack. Many members of the MAPK signaling cascade pathways are related to plant disease resistance, including defense gene activation, reactive oxygen species (ROS) generation, stomatal closure, phytoalexin biosynthesis, cell wall strengthening, and hypersensitive response (HR)-related cell death (Meng and Zhang, 2013), There may be crosstalk between each MAPK signaling cascade pathway and each member. DMGs in the MAPK signaling cascade were widely involved in nearly all stages of *B. elliptica* infection. DMGs including *MEKK1*, *MKK4/5*, *MKK3*, *MKK2*, *MPK3*, *MPK4*, *MPK6*, and *MPK1/2* were enriched in the MAPK pathway and were differentially expressed at different time points (Figure 5). Exogenous melatonin treatment increased MEKK1 gene expression. *MKK4/5* had the highest expressional level at 24 h respond to *B. elliptica* infection in both MB group and HB group, expression of MKK3 showed no significant difference in HB group, but exogenous melatonin treatment improved its expression at 0 and 24 h, and exogenous melatonin treatment increased MKK2 gene expression at 24 h after *B. elliptica* infection. The expression of MPK3 was significantly higher in the MB group than that in the HB group, *MPK4*, *MPK6*, and *MPK1/2* were differentially expressed during the *B. elliptica* infection process, and exogenous melatonin treatment increased gene expression at different time, respectively.

To confirm the reproducibility of the transcriptome data, 12 defense-related DEGs associated with the MAPK signaling pathway, plant–pathogen interaction pathway and plant hormone signal transduction pathway from the RNA-seq data were evaluated via qRT-PCR at 0, 12, 24, 36, and 72 h after infection according to their FPKM value. As shown in Supplementary Figure S3, the results of the qRT–PCR analyses were generally in accordance with the gene expression profile data from the transcriptome during the infection process. These results indicate good correlations between the transcription profiles and the RNA-seq data.

## 4 Discussion

### 4.1 Diverse Mechanisms of Plant Resistance to Pathogen Infection

Studies on the response of *Lilium* to *B. elliptica* infection have revealed that the plant defense response to pathogens is a complex biological process involving various changes at the cellular structure, physiological, biochemical and molecular levels. Studies on the molecular basis underlying resistance to *B. elliptica* have been performed, and on the basis of transcriptomic data, key genes involved in the jasmonate signaling pathway have been shown to play roles in certain plant species (*Lilium regale*) in defense against *B. elliptica*, whereas salicylic acid (SA) and ethylene (ET) were found to not be involved (Cui et al., 2018). The *Lilium* mRNA transcriptome revealed targets of miRNAs involved in metabolic processes (Gao et al., 2018), and lre-miR159a was shown to positively regulate resistance to *B. elliptica* and activate a defense response (Gao et al., 2020). In our study, the plant hormone signal transduction pathway and the phenylpropanoid and flavonoid pathways participated in the response of *Lilium* plants to pathogens at all stages of infection, according to KEGG analysis of different infection time points (Supplementary Table S5). This was similar to the findings reported by Nan Chai, in which Metabolic and transcriptomic analysis of *Lilium* plants infected with *B. elliptica* showed that differentially expressed genes (DEGs) and differentially accumulated metabolites (DAMs) were enriched in the phenylpropanoid and flavonoid pathways. Moreover, weighted gene coexpression network analysis (WGCNA) indicated that jasmonic acid (JA), SA, brassinolide (BR), and calcium ions (Ca^2+^) are important for defense against *B. elliptica* in *Lilium* (Chai et al., 2021). In addition, other plant hormones, including butyric acid (BA), BRs, and CKs, also play important roles in plant defense and immune responses (Glazebrook, 2005; Yang et al., 2015; Arnao and Hernandez-Ruiz, 2018). Recently, as reported with respect to the interactions between other hosts and pathogens, interactions between melatonin and plant hormones including ET, JA, SA, and ABA have been documented during pathogen infection (Zhao et al., 2021), but more research is needed for an in-depth understanding of the crosstalk between melatonin and plant hormone signaling pathways.

### 4.2 MAPK Cascade Is Involved in Plant Defense Induced by Melatonin

**FIGURE 6:**
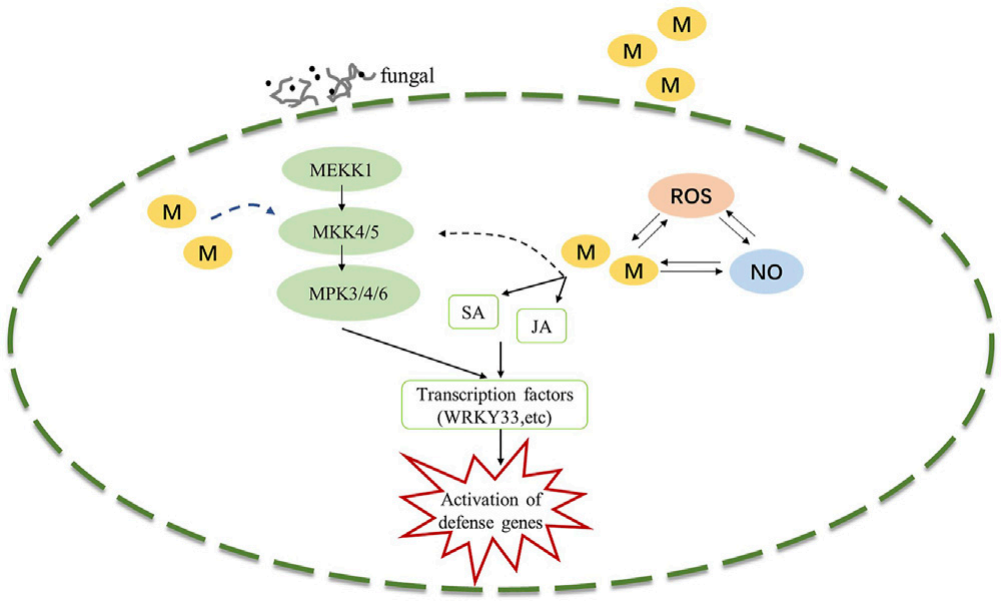
Potential model illustrating the melatonin induced defense signaling in *Lilium* against *B. elliptica*. *B. elliptica* infection or exogenous melatonin application induced activation of MAPK cascades and NO accumulation, which in turn activates defense related genes. Abbreviation: ROS, reactive oxygen species; MAPK, Mitogen-activated protein kinase; NO, nitric oxide; SA, salicylic acid; JA, jasmonic acid; M, melatonin.

The MAPK signaling cascade pathway participates in plant disease resistance by amplifying and transmitting foreign signals through phosphorylation cascades involving MAPK kinase kinase (MAPKKK, MEKK), MAPKK kinase (MAPKK, MEK), and MAPK proteins (Xu and Zhang, 2015; Thulasi Devendrakumar et al., 2018). There are many complex MAPK cascades involved in plant defense responses, such as *MEKK1*-*MKK4/MKK5*-*MPK3/MPK6* and *MEKK1*-*MKK1/MKK2*-*MPK4* cascades in *Arabidopsis thaliana* (Pecher et al., 2014; Peng et al., 2018), In our study, Exogenous melatonin treatment increased MEKK1 gene expression during the *B. elliptica* infection process. *MKK4/5* had the highest expressional level at 24 h respond to *B. elliptica* infection in both MB group and HB group, The expression of MPK3and MPK6 were significantly higher at 12, 24 and 36 h in MB group than that in HB group, We speculate that melatonin may play an important regulatory role via *MEKK1*-*MKK4/MKK5*-*MPK3/MPK6* in *Lilium* disease resistance to *B. elliptica*. A MAPK signaling cascade triggering melatonin-induced defense mechanisms via *OXI1/MAPKKK3*–*MAKK4/5/7/9*–*MAPK3/6* cascades has been elucidated, MAPKKK3 and oxidative signal-inducible 1 (OXI1) kinases play roles in triggering the melatonin-induced defense signaling pathway in Arabidopsis mutants, and MKK4/5/7/9-MPK3/6 cascades are responsible for melatonin-mediated innate immunity in MKK knockout Arabidopsis mutants (Lee et al., 2014; Lee and Back, 2016; Lee and Back, 2017). In our study, the DEGs involved in the MAPK pathway (*MEKK1, MKK4/5, MKK3, MKK2, MPK3, MPK4, MPK6, MPK1/2*) were differentially expressed between the MB group and the HB group according to the results of the qRT–PCR analyses and the RNA-seq data (Supplementary Figure S2), suggesting that melatonin was involved in the MAPK pathway and played a role in the resistance of *Lilium* to *B. elliptica*. According to the experimental results and those of previous studies, we speculate that melatonin-mediated stimulation of the MAPK kinase cascade reaction (*MAPKKK1*-*MAPKK4/5*-*MAPK3/6*) secondarily activates the transcription of the defense response (Figure 6). *MPK3*/*MPK6* phosphorylates *WRKY* transcription factors including *WRKY22*, *WRKY23*, *WRKY29*, *WRKY46* and *WRKY53*, which mediate the pathogen induced plant defense response (Yoo et al., 2014; Li et al., 2021; Xie et al., 2021). The study found that LrWRKYs may be important regulators involved in the biotic stress responses of lilies, the effects on plant immunity may result from the regulation of the SA-/JA-dependent signaling pathway (Cui et al., 2018; Fu et al., 2022). The mechanism of the MAPK signaling cascade-mediated plant defense response to pathogens has been gradually elucidated; this cascade mainly regulates transcriptional activation of defense genes, synthesis of plant antitoxins, cell wall thickening, hypersensitivity, stomatal closure, the production of endogenous hormones and ROS. However, the direct link between melatonin and the MAPK signaling cascades is unclear. Wei J. et al. (2018) found that the first phytomelatonin receptor (CAND2/PMTR1) in *A. thaliana*, CAND2/PMTR1, was induced by melatonin and acted on the α-subunit of heterotrimeric G proteins, which then activated NADPH oxidase (NOX) to produce H_2_O_2_ and promoted Ca^2+^ influx and K^+^ outflow, leading to stomatal closure (Wei J. et al., 2018). Nevertheless, does melatonin depend on the CAND2/PMTR1 receptor to convert extracellular signals into intracellular signals, activating intracellular signal transmission and regulating plant biological activities ? Answers to this question have not been reported. Many MAPK components are activated by melatonin (Zhao et al., 2021), but their specific pathways need to be further elucidated, which is also one of the key research goals in the future.

In conclusion, treatment with exogenous melatonin and infection with *B. elliptica* caused the differential expression of a large number of genes in *Lilium*. KEGG analysis showed that defense-related DEGs were mainly enriched in the plant–pathogen interaction pathway, plant hormone signal transduction, MAPK signaling pathway-plant, phenylpropanoid biosynthesis, and phenylalanine metabolism after melatonin treatment. The DEGs related to the MAPK pathway were significantly different between the MB group and the HB group, suggesting that melatonin may play a role in the disease resistance of *Lilium* to *B. elliptica.* Thus, plant resistance to fungi through the MAPK signaling cascade mediated by melatonin provides a new direction for fungal disease research.

## Data Availability

The datasets presented in this study can be found in online repositories. The names of the repository/repositories and accession number(s) can be found in the article/Supplementary Material.

## Supplementary Material

Supplementary Figure S1 Analysis of gene expression trend at 5 time point in melatonin pre-treatment group **(A)** and control group **(B)**.

Supplementary Figure S2 GO functional classification of differentially expressed genes.

Supplementary Figure S3 The qRT-PCR result and FPKM value of gene.

Supplementary Table S1 Quality statistics for transcriptome sequencing (RNA-seq) data.

Supplementary Table S2 The summary statistics of the assembled transcripts and unigenes.

Supplementary Table S3 The success rate of functional annotation in transcriptome unigenes.

Supplementary Table S4 Statistics of DEGs for GO enrichment analysis in different comparison group.

Supplementary Table S5 Statistics of DEGS for KEGG pathway enrichment analysis in different comparison group.

Supplementary Table S6 The sequence information of primers for RT-qPCR.
