# Supplementary figures and images for: Transcriptome Analysis Reveals that Exogenous Melatonin Confers Lilium Disease Resistance to Botrytis elliptica
Source: Front Genet. 2022 Jun 14;13:892674. doi: 10.3389/fgene.2022.892674 (PMC9237519; doi:10.3389/fgene.2022.892674)

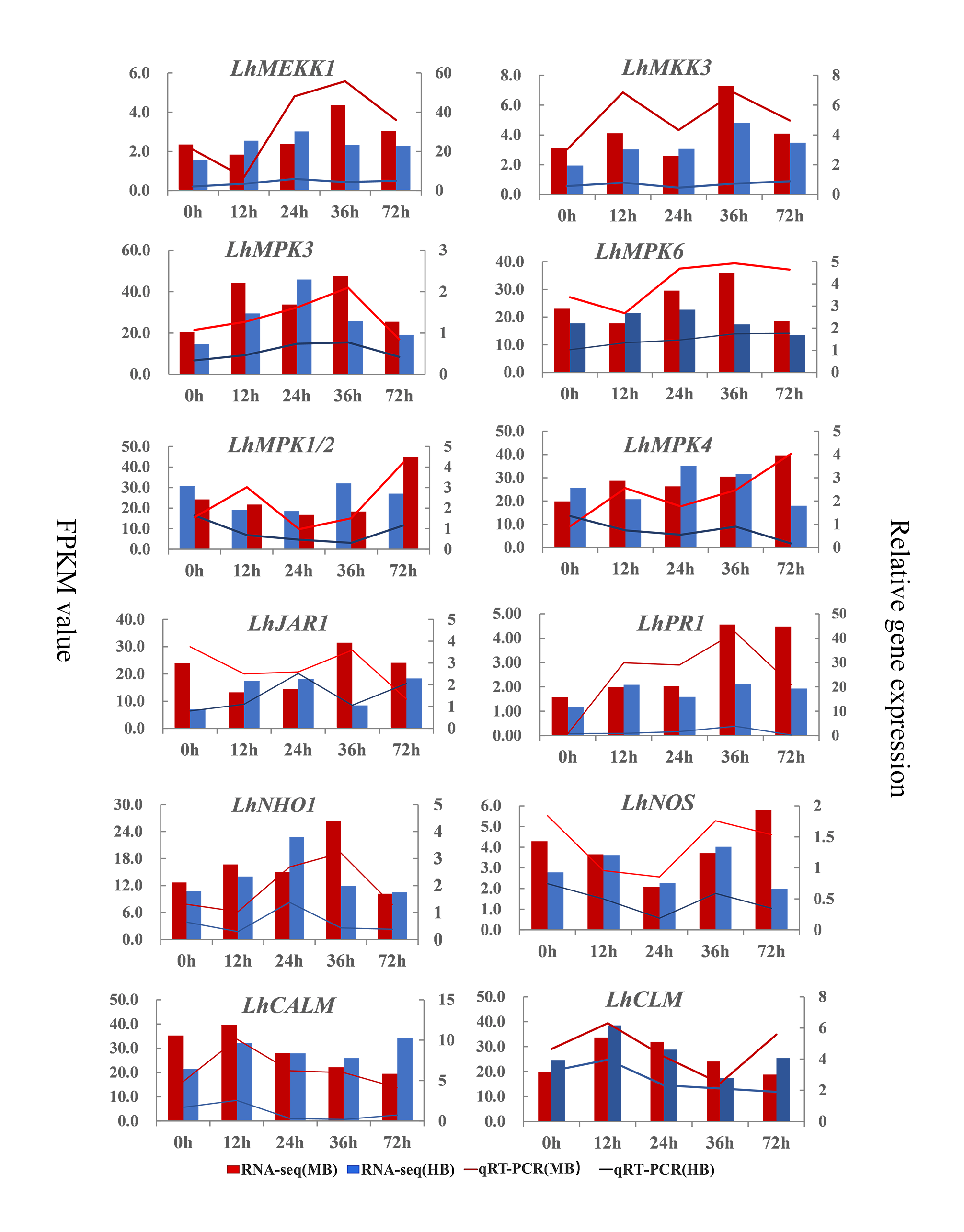

Supplement: Supplementary file 3 [file Image3.TIF]

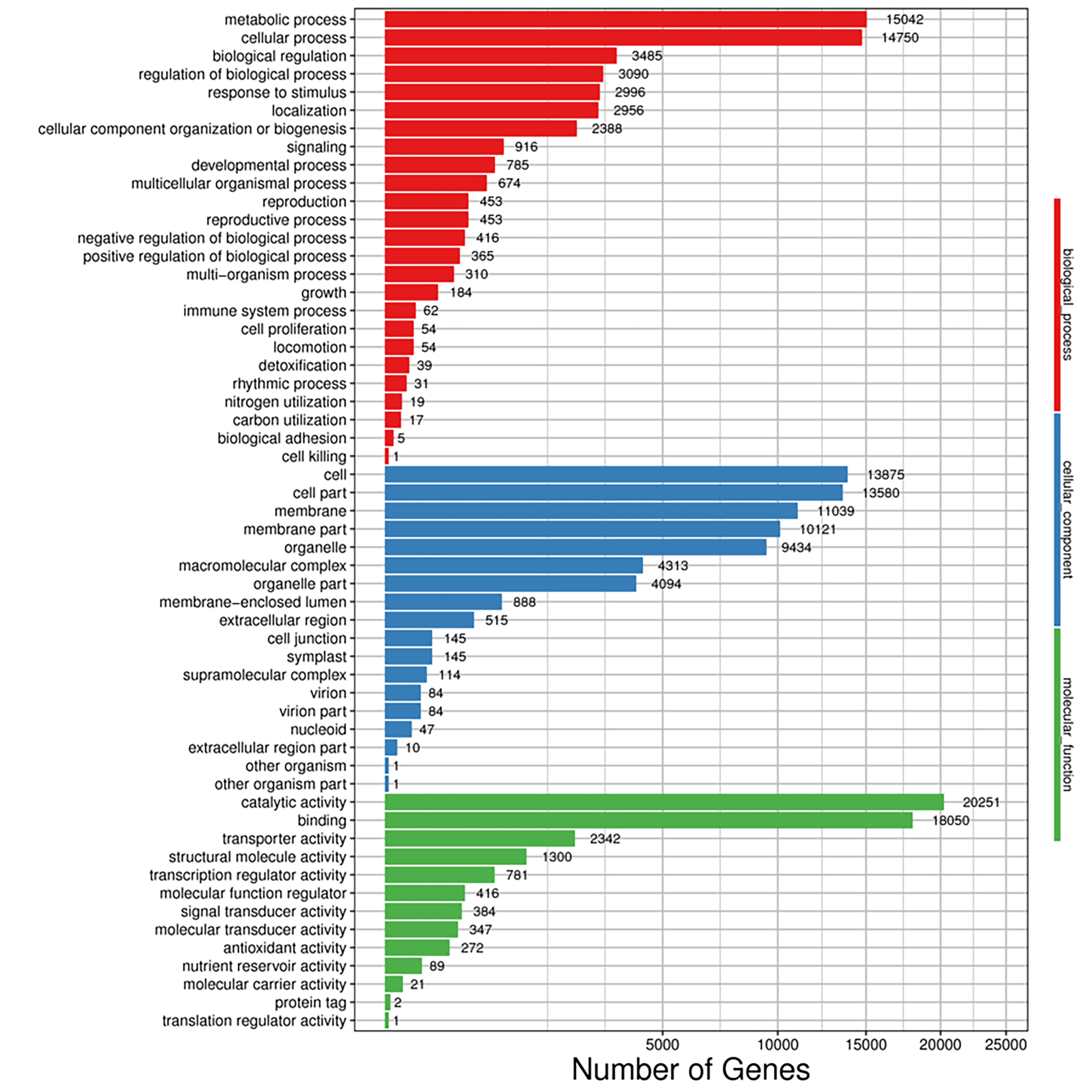

Supplement: Supplementary file 4 [file Image2.TIF]

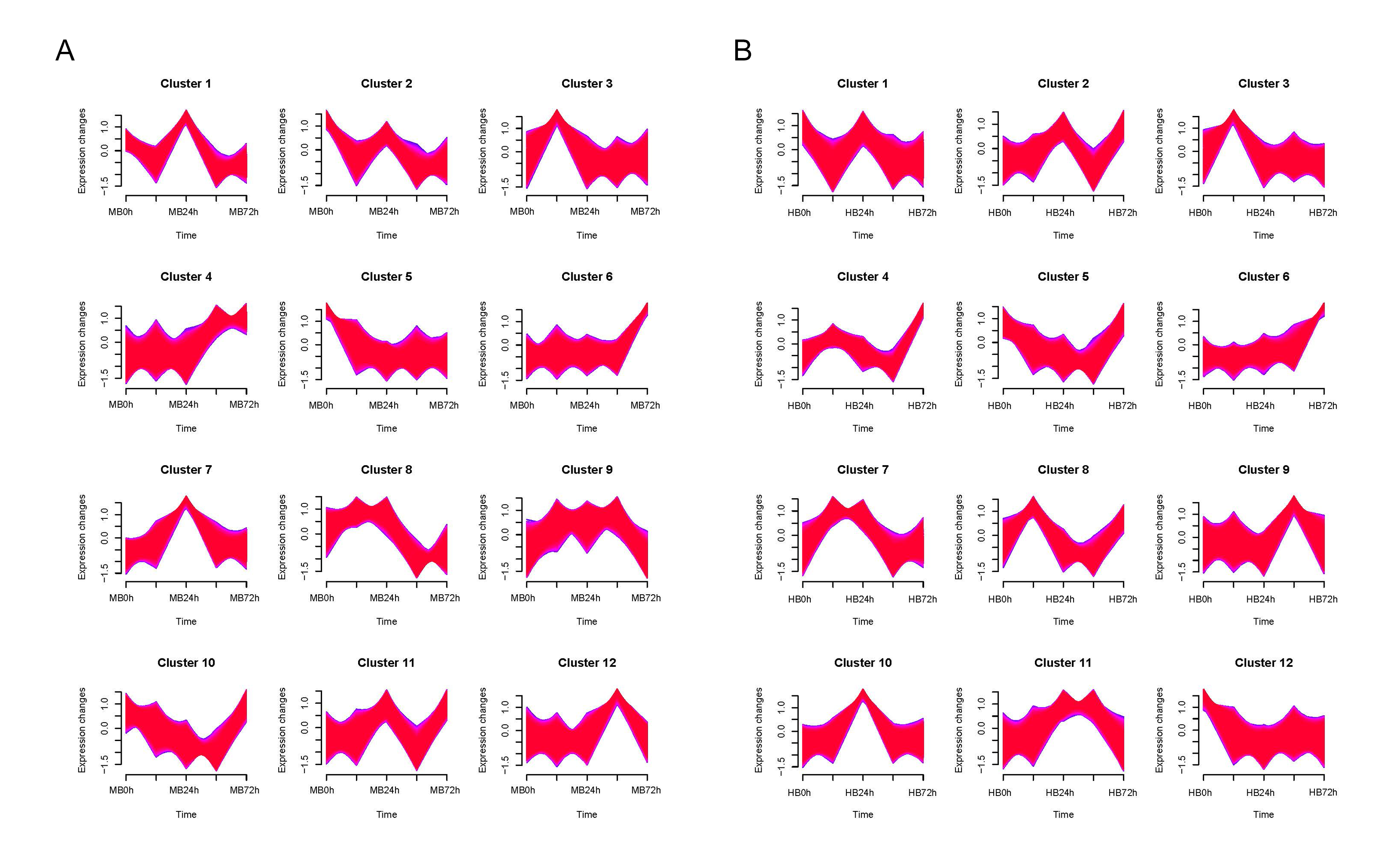

Supplement: Supplementary file 5 [file Image1.TIF]
