# Supplementary material for: Transcriptome Analysis Reveals that Exogenous Melatonin Confers Lilium Disease Resistance to Botrytis elliptica
Source: Front Genet. 2022 Jun 14;13:892674. doi: 10.3389/fgene.2022.892674 (PMC9237519; doi:10.3389/fgene.2022.892674)
Supplement: Supplementary file 8 [file Table6.DOCX]

### Table S6 Sequence information of primers for RT-qPCR

| Primer name | Primer sequence 5'-3' |
| --- | --- |
| Actin-F | TGTGCTTTCCCTCTACGCCAGT |
| Actin-R | TCCCTCACGATTTCCCGCTCT |
| MEKK1-F | TCATGCAAATGGGTCTATCAA |
| MEKK1-R | CTCTGGTGCCATCCAATATAC |
| MKK3-F | TGGAGCCTTGGACTAACT |
| MKK3-R | CTTTGGAGGTGTAGGTGATG |
| MPK3-F | CTCCGAGTTCATCACAGAACAG |
| MPK3-R | CGGTACCTCCAGTACAACATATTC |
| MPK6-F | TGATCCATGTGACGAAGCAG |
| MPK6-R | GAGCAGGTCGCGATTAAGAA |
| MPK4-F | GATTGATCACCGAGTTGATAGG |
| MPK4-R | GAACTGCTGCTTTGGATACT |
| MPK1/2-F | TCACAGGCGCTATCAAATG |
| MPK1/2-R | CCCAGGCTTCAAATCTCTATG |
| JAR1-F | GACAGCCACCTGTAACATATAC |
| JAR1-R | GGGACCGAATACAACTTCATC |
| NHO1-F | CGAGCGTCAATCAGTTCAT |
| NHO1-R | GCTTATCCCACTCCAGTTTC |
| NOS-F | CATGCACAGCCAGGTTTA |
| NOS-R | CCTTGGTTGGATTGCTGT |
| CALM-F | TCTTTGACAAGGACCAGAAC |
| CALM-R | CATCTCATCAACCTCCTCATC |
| CLM-F | CGAAGCATTCAGCCTCTT |
| CLM-R | TGGATTTGAGCTGGGATTG |
| PR1-F | GTGTCAGGCAACTCAATTTCTC |
| PR1-R | ACGACACTTACCTTGCACTC |
